# Cursive Eye-Writing With Smooth-Pursuit Eye-Movement Is Possible in Subjects With Amyotrophic Lateral Sclerosis

**DOI:** 10.3389/fnins.2019.00538

**Published:** 2019-05-29

**Authors:** Timothée Lenglet, Jonathan Mirault, Marie Veyrat-Masson, Aurélie Funkiewiez, Maria del Mar Amador, Gaelle Bruneteau, Nadine Le Forestier, Pierre-Francois Pradat, Francois Salachas, Yannick Vacher, Lucette Lacomblez, Jean Lorenceau

**Affiliations:** ^1^Département de Neurologie, Centre de Référence SLA-IdF, Hôpital Pitié Salpêtrière, AP-HP, Paris, France; ^2^Département de Neurophysiologie, Hôpital Pitié Salpêtrière, AP-HP, Paris, France; ^3^Laboratoire des Systèmes Perceptifs (UMR 8248), Département d’Études Cognitives de l’École Normale Supérieure, Paris, France; ^4^Institut du Cerveau et de la Moelle Epinière (ICM), UMRS 975, ICM-INSERM 1127, FrontLab, Paris, France; ^5^Département de Neurologie, Institut de la Mémoire et de la Maladie d’Alzheimer, Centre de Référence National ‘Démences Rares’, Hôpital Pitié Salpêtrière, AP-HP, Paris, France; ^6^Centre de Recherche en Myologie, UMRS974, Equipe 10 NMCONNECT, Sorbonne Université, Paris, France; ^7^Département de Recherche en Éthique, EA 1610: Etude des Sciences et Techniques, Université Paris Sud/Paris Saclay, Paris, France; ^8^Sorbonne Université, CNRS, INSERM, Laboratoire d’Imagerie Biomédicale, Paris, France; ^9^Northern Ireland Centre for Stratified Medicine, Biomedical Sciences Research Institute Ulster University, C-TRIC, Altnagelvin Area Hospital, Londonderry, United Kingdom; ^10^Délégation à la Recherche Clinique et à l’Innovation (DRCI), Hôpital Saint-Louis, APHP, Paris, France; ^11^Sorbonne Université, INSERM UMRS 1127 and CIC-1422, ICM, Hôpital Pitié Salpêtrière, Département de Neurologie, AP-HP, Paris, France; ^12^Sorbonne Université, Institut de la Vision, Inserm UMR S 968-CNRS UMR 7210, Paris, France

**Keywords:** amyotrophic lateral sclerosis, assisted communication devices, smooth-pursuit eye movements, pilot clinical study, motor learning

## Abstract

Amyotrophic lateral sclerosis (ALS) is a neurodegenerative disorder causing a progressive motor weakness of all voluntary muscles, whose progression challenges communication modalities such as handwriting or speech. The current study investigated whether ALS subjects can use Eye-On-Line (EOL), a novel eye-operated communication device allowing, after training, to voluntarily control smooth-pursuit eye-movements (SPEM) so as to eye-write in cursive. To that aim, ALS participants (*n* = 12) with preserved eye-movements but impaired handwriting were trained during six on-site visits. The primary outcome of the study was the recognition of eye-written digits (0–9) from ALS and healthy control subjects by naïve “readers.” Changes in oculomotor performance and the safety of EOL were also evaluated. At the end of the program, 69.4% of the eye-written digits from 11 ALS subjects were recognized by naïve readers, similar to the 67.3% found for eye-written digits from controls participants, with however, large inter-individual differences in both groups of “writers.” Training with EOL was associated with a transient fatigue leading one ALS subject to drop out the study at the fifth visit. Otherwise, itching eyes was the most common adverse event (3 subjects). This study shows that, despite the impact of ALS on the motor system, most ALS participants could improve their mastering of eye-movements, so as to produce recognizable eye-written digits, although the eye-traces sometimes needed smoothing to ease digit legibility from both ALS subjects and control participants. The capability to endogenously and voluntarily generate eye-traces using EOL brings a novel way to communicate for disabled individuals, allowing creative personal and emotional expression.

## Introduction

Amyotrophic lateral sclerosis (ALS) is a neurodegenerative disorder causing a progressive motor weakness of all voluntary muscles, with notable exceptions such as extra-ocular muscles that remain durably spared (Brockington et al., 2013; Gorges et al., 2015; Nijssen et al., 2017). Its progression challenges communication modalities such as handwriting or speech with a marked impairment of quality of life for patients and caregivers. As there is no cure, treating symptoms and disability remains of major importance, and recent recommendations for clinical management in ALS indicate that patient autonomy and ability to communicate should be promoted (Andersen et al., 2012). In recent years, the development of appropriate assistive communication devices (ACD), including those controlled by eye-movements, played a key role to maintain patients in an efficient interaction with their environment and caregivers, resulting in a positive impact on quality of life (Münßinger et al., 2010; Caligari et al., 2013). There is, however, an unmet need to personalize communication and provide more creative tools relying on the actions made by the participants.

As a matter of facts, classical eye-controlled ACD are based on the triad “saccade, fixation, selection” of predefined items (e.g., letters of the alphabet) displayed on a computer screen. These items constrain the choices of the user, who cannot generate figures or symbols of its own, thus limiting its creativity. This study evaluates whether ALS subjects can use a new eye-operated system (Eye-On-Line, EOL)^1^ that relies on the voluntarily control over smooth-pursuit eye-movements (SPEM) (Krauzlis, 2005; Lorenceau, 2012). Using EOL, subjects face a temporally modulated visual display eliciting an illusory perception of motion that provides a *positive* visual feedback to the oculomotor system. Relying on this feedback, individuals can gain volitional control over SPEM. After a training period (2–10 sessions of 30 min), subjects can generate smooth renderings of digits, letters, words or drawings at will (Lorenceau, 2012). Eye-written letters and words endogenously generated with EOL are similar to the subject’s hand-writing (Lorenceau, 2012), who can identify herself/himself with their actions -including writing their own signature- that reflect his/her emotions and personality.

To our knowledge, using SPEM to eye-write in cursive with healthy participants was first described in a recent article showing the large variety of eye-written production that can be achieved (Lorenceau, 2012). Whether EOL can be used by ALS participants was the primary goal of this study. Learning to master one’s SPEM would provide evidence that new (oculo-) motor skills can be acquired by ALS subjects despite their disease, and could bring a novel way of communication. EOL was first tested on 20 healthy participants to establish a stepwise training program designed to: (1) optimize the EOL display parameters that elicit a perception of illusory motion induced by eye-movements, detailed thereafter; (2) train participants to initiate and maintain SPEM for long durations; (3) execute imposed motor plans of increasing complexity (lines, figures, digits, letters, and words). Using the outcomes of this preliminary study, we evaluated the feasibility and safety of EOL with ALS subjects.

## Methods

### Study Design

Participants were recruited from the Department of Neurology at the Pitié-Salpêtrière Hospital with the following inclusion criteria: (1) probable or definite ALS disease according to the revised El Escorial criteria; (2) impaired handwriting but still intelligible speech; (3) absence of oculomotor impairment. Subjects with a history of epilepsia, with clinical evidence of oculomotor impairment, or with dementia were excluded. The study was carried out in accordance with the Declaration of Helsinki and was approved by all relevant ethics committees and national regulatory authorities (CPP n°14942 IDF V; ANSM 2014-A00392-45). All participants gave informed consent (written consent whenever it was possible). The study (recorded on Clinical Trials reference NCT02313402)^2^.

### Participants

Between May 2014 and April 2015, twelve ALS subjects (3 females, 9 males) were enrolled: 8 participants with spinal ALS and 4 participants with a bulbar form. Their mean age was 56.8 years old, with an average disease duration of 43.3 months (SD: 42.4) and mean ALSFRS-R score of 35 (SD: 6.6). Table 1 presents the details of clinical data. One (patient A) left the study at the fifth visit. We however included the patient’s data, whenever it was relevant.

**Table 1:** Clinical characteristics of the ALS participants.

Patient No.	Age (years)	Sex	Disease duration (months)	Site of onset of ALS	ALSFRS score	Forced vital capacity
A	62	M	33	UL	32	108
B	66	M	61	LL	28	63
C	51	F	31	LL	43	113
D	61	F	25	B	31	24
E	49	M	41	B	23	Impracticable
F	50	M	32	LL	28	55
G	66	M	36	UL	45	122
H	71	M	34	LL	38	134
I	74	M	8	UL	39	66
J	20	M	11	LL	38	77
K	59	M	37	B	37	114
L	53	F	171	LL	38	93
Mean (*SD*)	56.8 (14.2)	–	43.3 (42.4)	-	35 (6.6)	88.1 (33.8)


*M, male; F, female; UL, upper limbs; LL, lower limbs; B, bulbar.*

### Intervention

**FIGURE 1:**
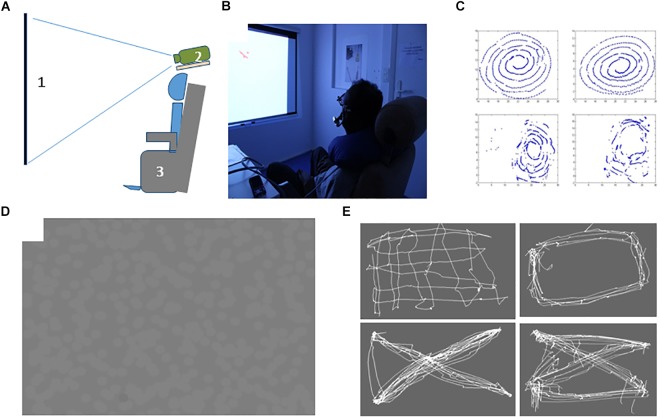
Overall display settings. Participants sat on a comfortable chair with armrests and headrest, in front of a large video screen. To minimize measurement errors, head movements were restrained using a necklace ergonomic cushion. **(A)** Schematic view of the display: 1, 2 Screen and video projector used to display the stimuli; 3. Adjustable chair for the participants. **(B)** Photograph of the settings used in the study. **(C)** Examples of accurate and poor SPEM produced during tracking of a visible target undergoing a spiral motion. **(D)** EOL stimulus made of 300 randomly distributed disks whose contrast polarity changed at ∼10 Hz on a uniform background. **(E)** Top: Examples of freely generated eye-movements from ALS participants after training with EOL. See text for details.

The EOL training program comprised six sessions (two sessions per week for 3 weeks with a minimum break of 1 day between sessions). Each session lasted at most 2 h, including breaks and pauses. ALS subjects initially (at inclusion during Session 1) underwent behavioral and neuropsychological assessments, including the hospital anxiety and depression scale (HAD, Zigmond and Snaith, 1983), the Frontal Behavioral Inventory scale (FBI, Kertesz et al., 1997), the Starkstein apathy scale (Starkstein et al., 1992), the Mattis Dementia Rating Scale (MDRS, Lavoie et al., 2013), and two tests (identification of emotion and theory of mind) from the social cognition and emotional assessment (SEA, Funkiewiez et al., 2012). Fatigue and motivation were quoted with a visual analogical scale (VAS) at the beginning and at the end of each session (from 0: “I have no motivation”/“I am not tired,” to 100: “I have a great motivation”/“I am extremely tired”). Safety was determined by a systematic inventory of adverse events occurring during or between on-site visits. The first contact with the EOL display and the principle of cursive eye-writing was introduced at the second visit, and training unfolded until the sixth visit. During training, subjects sat on a comfortable chair with armrests and headrest, placed at 180 cm from a video screen (Figure 1A,B). To minimize measurement errors, head movements were restrained using a necklace ergonomic cushion. The visual stimulus used for eye-writing was displayed with a video projector (Epson EH-TW480, 60 Hz).

#### EOL Device and Principle

Details about the principles underlying EOL can be found in Lorenceau (2012), and examples of cursive eye-writing and drawing are available at *http://eol.scicog.fr/demo/demo_ang.html*. Briefly, EOL relies on the finding (Anstis, 1970) that alternating the sign of contrast of a moving stimulus between 8 and 20 Hz changes the perceived direction of motion, an illusion called “reverse-phi” motion. This manipulation fools the cortical neurons selective to motion direction that invert their direction preference with alternating contrast polarity as compared to constant contrast polarity (Krekelberg and Albright, 2005). This appears to be an intrinsic property of these neurons captured by models of motion selective neurons (Adelson and Bergen, 1985). When observing a *static* stimulus alternating its contrast polarity, the retinal slip accompanying eye-movements, and SPEM in particular, is a physical retinal stimulus moving in a direction *opposite* to that of the eyes, but thanks to the “reverse-phi” illusion, the *perceived* motion associated to the retinal slip is in the *same* direction as the eyes.

The stimulus against which subjects learned to generate SPEM consisted in 300 identical static disks (1° of visual angle), randomly distributed on a gray background (24 cd/m^2^). All disks had identical luminance, but the contrast alternated periodically, switching from light-to-dark and dark-to-light relative to the background at about 10 Hz. Moving the eyes while looking at this display elicits a retinal slip of the disks in a direction *opposite* to the eyes’ direction. Nevertheless the whole screen appears as a full-field flow of blurry disks *seemingly moving in* *the* *same* *direction* *as the eyes*, thus reproducing the movements of the eyes. This *eye-induced illusory motion* is well documented, but describing the underlying mechanisms is out of the scope of this article (see Anstis, 1970; Krekelberg and Albright, 2005; Lorenceau, 2012). When perceived and attended, this eye-induced illusory motion provides an on-line visual feedback on one’s own eye-movements that can be used as a moving visual substrate to voluntary initiate and sustain SPEM in any direction: moving the eyes perpetuates the illusory motion that in turn feeds the oculomotor system, establishing a positive visuo-motor feedback loop. Training is necessary to master this loop, and unfold in different steps: (1) the eye-induced illusory motion must first be perceived and attended; (2) once the illusory motion becomes familiar, one can generate short episodes of SPEM; (3) after these initial steps, it becomes possible to voluntary generate figures such as circle, ellipse, lines, etc, using SPEM; (4) Finally, participants can project the over-learned motor plans corresponding to the cursive writing of digits, letters or words. Note that the eye-movements are not explicitly drawn as graphic traces on the screen, but are nevertheless seen by the “eye-writer” as a faint wake produced by the retinal slip. The ease with which participants generate SPEM depends on the stimulus characteristics, such as the contrast of the disks, and the frequency of light-to-dark and dark-to light alternations (Portron and Lorenceau, 2017). During the training sessions, these parameters were interactively adjusted depending on the participants’ report of seeing an illusory motion during their eye-movements. In general, the temporal frequency ranged between 10 and 15 Hz. The contrast was lowered as much as possible to avoid discomfort and fatigue, while still maintaining a faint perception of illusory motion during eye-movements production.

### Material, Oculomotor Tests, and Training Procedure

After a 5-points eye-tracking calibration procedure, the movements of the right eye were recorded with a head-mounted infrared video-based eye-tracker (EyeTechSensor, sampling rate 60 Hz, *Pertech*^^®^^), using the position of the pupil over time. To provide a reference base line, each session started with basic oculomotor tasks. The proper training with the EOL device started at session 2 and lasted until session 6, with task difficulty adapted to the progress of each participant.

*Fixation task*: Subjects were asked to keep fixating a target displayed in the center of the screen for 3.3 s. The time spent in a predefined reference window (2° of visual angle) was used to assess the quality of fixational eye movements.

*Smooth-pursuit task:* Subjects were asked to track a visible moving disk, starting from the center position and describing a spiral trajectory with increasing speed (from 0 to 0.879 m/s), until it reached the zenith point 40 cm above the starting position after 20 s of motion (see Figure 1C). The quality of pursuit was estimated by counting the number of catch-up saccades and by computing the pursuit gain (the ratio of eye-speed to target speed).

#### Training With the EOL Device

After the first contact with EOL in session 1, subjects’ eye-movements were repeatedly recorded during short episodes with the EOL display (Figure 1D), each lasting 30 to about 60 s. The different runs were separated by short breaks allowing participants to rest, a time used to collect their reports on their feelings, their perception of motion and their subjective appreciation of their eye-movements. After each recording, participants were shown their eye-traces, rendered as dots or line-drawings, so that they could appreciate whether they were producing saccades and/or SPEM. The contrast of the disks was initially high (30–60%), and progressively lowered depending on the participants’ reports and the smoothness of the eye-traces, so as to avoid fatigue and discomfort and to minimize the salience of static position cues provided by the disk borders.

During a session, participants performed between 5 and 15 short interactive runs with advices and instructions to help them perceiving the illusory motion and to induce and maintain SPEM (e.g., by moving their head, or waving their hand in front of the EOL display). Whenever participants succeeded to perceive the illusory motion, they were guided to generate SPEM, either by maintaining it as long as possible, or by executing specific figures. Afterward, subjects performed 5–10 runs on their own, and were free to execute eye-movements as they wanted (Figure 1E), but were initially oriented to restrain to simple movements, as drawing lines or circles, and later to produce more complex figures (waves, bridges, spirals, digits and letters). The production of digits was introduced when subjects could produce and maintain SPEM at will for long durations (>500 ms), or to draw complex figures, but no later than the fourth session. In the following, we first present SPEM produced during runs without feedback, and then present the digit recognition experiment.

### Eye-Data Analyses

All eye-data were processed off-line with Matlab (version 2017a, The Mathworks). The different oculomotor tests (fixation, smooth-pursuit gain) were analyzed for each participant and each session. We used Linear Mixed Effects Model to analyze all the parameters derived from eye-movements, with subjects as crossed random effects (including by-subject random intercepts, see Baayen et al., 2008). The models were fitted with the lmer function from the lme4 package (Bates et al., 2014) in the R statistical computing environment (version 3.3.1). We report regression coefficients (b), standard errors (SE) and *t*-values. Fixed effects were deemed reliable if |t| > 1.96 (Baayen, 2008). We mostly focused on training effects, comparing the first-half sessions (FH sessions 2–3) to the second-half sessions (SH sessions 5–6) of the training sessions. We used the values from FH as references for the LMEs.

#### Quantification of Freely Generated Smooth-Pursuit With the EOL Device

For each recorded run of each subject, saccadic eye-movements were detected using both a velocity and an acceleration threshold (50°/s, 500°/s^2^, respectively). Data corresponding to blinks (pupil size equal to zero), were detected and removed from further analyses. Smooth pursuit was defined as any eye-trace comprising at least a cluster of 30 successive points (corresponding to 500 ms of recording) spaced by less than 50 pixels -to avoid including saccadic eye-movements-, and whose standard deviation was larger than 10 -to avoid including fixational eye-movements in the quantification of pursuit. The longest pursuit and the cumulated duration of pursuit (averaged and best runs) were determined for each run of each session and used throughout the study to evaluate the capability to generate smooth-pursuit at will.

#### Outcomes Measures

To address the issue of using the EOL device to communicate, participants were requested to “eye-write” digits from 0 to 9. The generation of eye-written digits started no later than session 4 and was repeated until the end of the training program. The traces recorded in session 6 were collected from all subjects and processed to isolate each digit, stored as a separate graph. In addition to the raw traces, we generated smoothed versions of each digits, using a sliding averaged (width of the sliding window = 6 samples). We similarly, processed digits generated by twelve healthy controls matched for age and gender who followed the same training protocol. The eye-written digits of ALS and control subjects were shuffled, resulting in 440 digits (10 digits × 2 versions × 22 eye-writers). These digits were presented in random order on a computer screen to 20 naïve “readers” who were asked to identify each digit by entering their response with a keypad (0–9), and to respond at random whenever this appeared too difficult or impossible. The identification test lasted around 15 min. The correct identification rates were measured for ALS and control subjects with both the raw and smoothed traces.

## Results

### Eye-Movements

The recorded eye-movements were highly variable within and across participants and sessions, both in the basic tests made at the beginning of each session (quality of fixation, ability to track a moving target), as well as for the EOL runs. These differences, observed for the ALS and control subjects, may reflect day-to-day modulations of cognitive states (attention, concentration, willingness to perform eye-movements). This intra and inter individual variability, together with the large data set collected over sessions, precluded conducting fine statistical analyzes of all eye-movement parameters at the group level on a session-by session basis. In the following, we focus on the mean duration of endogenously generated smooth-pursuit, and the longest pursuit realized during the different sessions. To assess the effect of training and to determine whether subjects made progress over sessions, we made statistical tests using the SPEM parameters of the early and late sessions. We then present the results of a separate experiment where “naïve” readers had to identify eye-written digits (see below), which is most relevant to evaluate the outcomes of the study and the feasibility of eye-writing for communication.

#### Quality of Fixation and Target Tracking

To analyze the effects of training on fixation and target tracking, we compared the mean results from sessions 2 and 3 (FH) to the sessions 5 and 6 (SH). The standard-deviations of the horizontal and vertical eye-positions in the fixation test averaged across participants are presented in Supplementary Table 1. Overall the quality of fixation was similar at the group level during early and late sessions (*b* = -0.78; *SE* = 3.80; *t* = -0.20).

The average pursuit gains during target tracking (see section 2.5) measuring the quality of SPEM are reported in Supplementary Table 1. Overall the SPEM gains did not significantly change between the early and late sessions at the group level (*b* = -0.0029; *SE* = 0.0027; *t* = -1.05).

Although these averaged data indicated that training had no net effect on the quality of fixation or of SPEM at the group level, individual results may differ, as indicated by the large standard deviations of the results.

#### Volitional Generation of Smooth Pursuit With EOL

**FIGURE 2:**
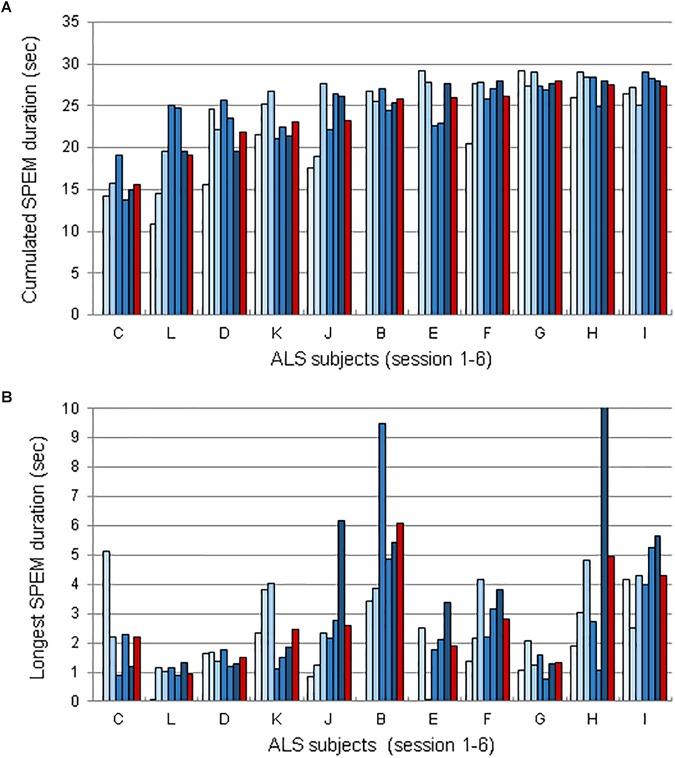
Duration of SPEM voluntarily generated SPEM using EOL, for each ALS subject during sessions 1–6, sorted from lowest to highest mean performance. **(A)** Mean cumulated duration of SPEM durations per session. **(B)** Longest duration of voluntarily generated SPEM for each session and participant. Red bars represent the averaged across sessions. Note that 3 subjects (C, B, and E did not use EOL in session 1).

Examples of eye-movements generated at will during the EOL training sessions are shown in Figure 1E. To determine whether participants progressively mastered SPEM with the EOL device, we detected episodes of SPEM using the criteria defined above (see section 2.6.1.). We then computed the cumulated duration of episodes of SPEM, the longest continuous SPEM, and the best run during which participants attempted to endogenously generate SPEM. Overall, comparing the average percentage of SPEM (% of the time spent making SPEM) and the best values amongst these runs in FH and SH sessions revealed no effect of training (Average: *b* = 1.03; *SE* = 2.61; *t* = 0.39 | Best: *b* = 3.90; *SE* = 3.50; *t* = 1.11; Supplementary Table 2). However, the inter-individual differences were large, with some participants producing short episodes of SPEM, while others were able to generate SPEM at will for long durations. Moreover, performance was not continuous, with ups and downs across or within sessions. To determine whether some participants progressively learned to master their SPEM, we analyzed in more details the time during which each participant generated SPEM, as well as the longest duration of SPEM (Figure 2). Note that we could not record SPEM with EOL during session 1 with patients C, B and E whose results therefore comprise only 5 bars (one per session, plus the average, shown by a red bar).

Figure 2 presents SPEM data for each participant and each session. Figure 2A represents the time spent making SPEM during a run. Each bar of the histograms (one histogram per subject) corresponds to one session; the red bar is the average of the runs across sessions for each subject. Figure 2B represents the longest SPEM produced during a run, using the same representation as Figure 2A. As it can be seen, 8 subjects were able to generate SPEM for more than 2 s, which considerably outperform the duration of SPEM that can be produced in absence of the EOL device (i.e., ∼250 ms on a uniform background, see Lisberger et al., 1987).

**FIGURE 3:**
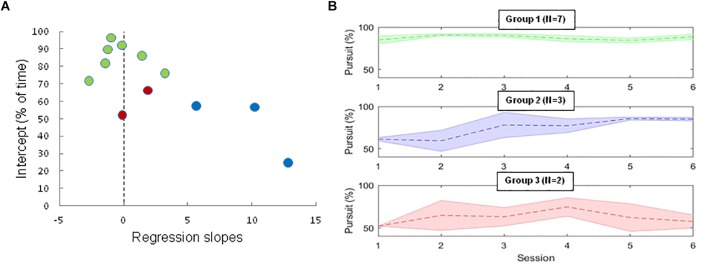
**(A)** Coefficients of linear regressions (intercepts as a function slope) computed on the duration of SPEM in the best EOL runs of each session three groups can be distinguished: 1. ALS subjects with a high intercept (>70) and low slope (<3) already performing well at session 1, with little improvement over sessions (green symbols); 2. ALS subjects with a low intercept (<60) and a steep slope (>5) indicating these patients made progress over sessions (blue symbols); 3. ALS subjects with low intercept (<70) and low slopes (<3) corresponding to patients who performed poorly and did not improve over sessions (red symbols). **(B)** Percentage of time spent producing SPEM across sessions for the 11 ALS subjects. (Top green) participants quickly mastering SPEM. (Middle, blue) Participants increasing the time spent producing smooth-pursuit over sessions. (Bottom, red) Participants failing to generate SPEM at the end of the training period.

To get insights into the highest level that ALS participants can reach, we took the best runs for each session and each subject, corresponding to the longest cumulated duration of SPEM production in a session, expressed in percentage of the run duration. We then computed a linear regression on these data to assess the initial best performance (Intercept) and the learning rate (regression slopes) of each participant. The intercepts and the slopes computed in this way are plotted in Figure 3A for all ALS subjects.

A high intercept indicates participants that were already performing well at the beginning of the training program, while low intercepts denote participants who performed poorly at start. Further, large slopes indicate a progression during training, while small (or negative) slopes indicate no progress over sessions. As it can be seen, seven ALS subjects initially performed well (producing SPEM for more than 70% of the time during a 30 s run, green symbols), and therefore could not improve much over sessions (ceiling effect resulting in small regression slopes). Three subjects were initially not performing well (low intercept), but made progress over sessions (steep learning slopes, Blue symbols). Finally, two subjects were initially not performing well (low intercept) and did not improve over session (small or negative slopes, Red symbols). Note, however, that task difficulty increased over sessions (from freely generating SPEM to voluntarily producing specific figures), such that the reported lack of improvement must be considered with caution.

This pattern of results suggests the existence of different profiles, as shown in Figure 3B: (1) Participants who initially failed to perceive the EOL motion illusion, and only produced short episodes of SPEM (*N* = 2; patients A, B). Despite training, these participants produced series of small saccades instead of sustainable smooth-pursuit. (2) Participants who gradually improved their capability to produce SPEM at will (*N* = 3; subjects C, J, L). (3) Participants who were able to successfully produced SPEM at the beginning of the training sessions, and whose mastering of EOL remained stable throughout the study (*N* = 7; subjects D, E F, G, H, I, K). This pattern of interindividual differences is similar to that found with control subjects (data not shown; see Lorenceau, 2012).

#### Production and Recognition of Eye-Written Digits

**FIGURE 4:**
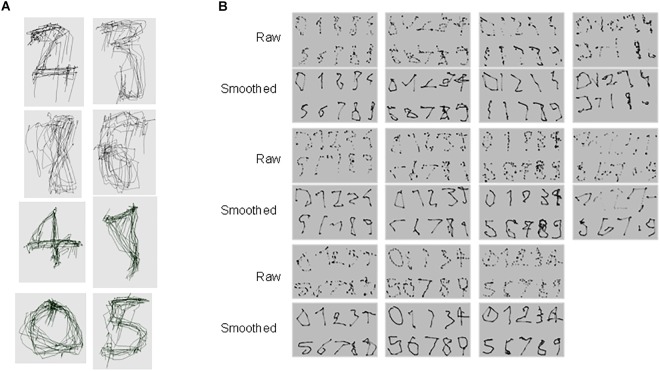
Digits eye-written by ALS subjects during session 6. **(A)** Examples of digits produced in succession, separated by blinks, during a single 30 s run. The eye-traces were segmented off-line to isolate one exemplar of each digit for each subject. **(B)** Raw and smoothed eye-traces (sliding averaged, *n* = 6 samples) were used in the experiment. Digits generated by ALS and control subjects, computed in the same way, were randomly mixed.

**FIGURE 5:**
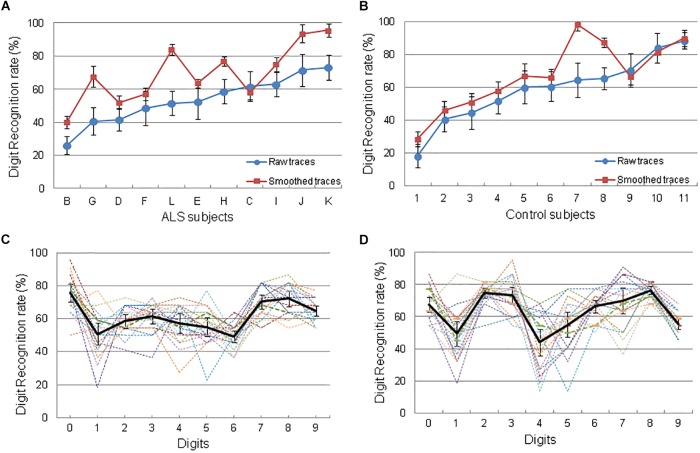
Results of the digit identification experiment performed by 22 “naïve” readers. 440 digits from ALS and control subjects (10 digits × 2 Versions, × 22 subjects) were mixed and randomly presented in succession on a computer screen. Readers identified each digit (from 0 to 9), or responded at random when unable to read a digit. **(A)** Left: identification rate of digits generated by ALS subjects for smoothed (red symbols) and raw (blue) eye-traces. **(B)** Right, identification rate of digits from the control subjects. Colors as in A. Error bars represent 1 SD computed over the readers. **(C,D)** Recognition rate for each of the 10 digits by each reader (colored lines) and averaged recognition rate (black line), for the ALS (left) and the control (right) groups. Error bars represent 1 SD computed over the readers.

To evaluate the legibility of eye-written digits, we tested how well “naïve” readers (*N* = 20) could identify the digits produced during session 6 (see section 2.6.2; Figure 4B). During this session, all subjects eye-wrote a digit between 5 and 12 times in a row during 30 s runs (see examples in Figure 4A), indicating that ALS subjects were able to write digits at will. Each digit (between 0 and 9) was written several times in separate runs. We then selected an exemplar amongst all eye-written digits for the recognition experiment, and used either the raw trace or a smoothed version of each trace (Figure 4B). The same procedure was used with control subjects. The recognition rates of the “readers” (Figure 5A,B) are very similar for ALS and control participants. The readers recognized the digits with similar scores with, on average, a recognition rate of 69.4% for ALS subjects and of 67.3% for control subjects with smoothed digits, with identification rates ranging from ∼25 to 95%, depending on the participant. To determine whether some digits were more difficult to write –and/or to read- we analyzed the recognition rate for each digit for all the “readers” (Figure 5C,D). As it can be seen in Figure 5C,D, some digits (1 and 4 in particular) were more difficult to read, or led to confusions, as indicated by the larger variability amongst the readers for these digits.

**FIGURE 6:**
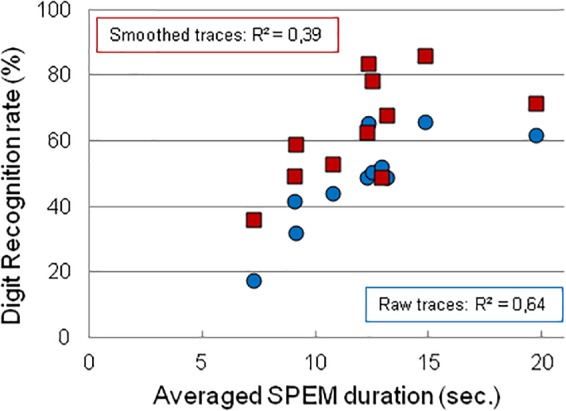
Correlation between the averaged duration of freely generated SPEM and the recognition of digits eye-written by ALS participants, either in their raw version (Blue dots) or in their smoothed version (Red squares). Subjects able to sustain SPEM for long durations are more likely to generate recognizable digits.

We then evaluated whether the capability of ALS subjects to produce long SPEM during training was correlated with the recognition rate of the digits (Figure 6). We found a modest correlation between the averaged SPEM duration and the recognition rate of *raw* eye-written digits (*R*^2^ = 0.64), while the correlation between the averaged SPEM duration and the recognition rate of *smoothed* eye-written digits was weaker (*R*^2^ = 0.39). This suggests that the ALS subjects able to sustain SPEM for a long duration were also those able to directly write recognizable digits, although smoothing often improved legibility.

### VAS Scale

Averaged tiredness over the training program was 26.15 (SD 25.6); averaged motivation was 87.35 (SD 16.16). To determine the evolution of these features over time, we compared the results from the FH sessions (2, 3) to the SH sessions (5, 6). The results (Supplementary Table 3), revealed no evolution of tiredness (*b* = 1.82; *SE* = 3.25; *t* = 0.56), and motivation (*b* = 2.71; *SE* = 2.69; *t* = 1.00).

### Neuropsychological Tests and Inter-Individual Eye-Writing Performance

Results from neuropsychological tests are detailed in Supplementary Table 4. Although large inter-individual differences in eye-movement control are found in the general population^15^, we tried to determine whether the performance of ALS subjects was related to behavioral or neuropsychological scores of the tests administrated during session 1. Despite the fact that the small sample size precludes any firm conclusion, we expected that a systematic dependence of eye-movement performance on specific traits could emerge and point toward an origin of the observed differences. However, no salient relationships between the different scores and eye-movements, or with age or gender, were observed at the group level. We thus analyzed individual results to evaluate possible indications that could account for the EOL results. We found that only one subject (patient A) had global cognitive impairment according to MDRS, in addition to abnormal social cognition (emotion and theory of mind) and depression. Four subjects (patients A, B, J, L) were considered as apathetic, in association with frontal behavioral disorder for patient B.

### Safety and Adverse Events

No serious adverse event occurred during the study. Using EOL was associated with accentuated fatigue on VAS and one ALS subject dropped-out at fifth visit because of excessive tiredness and cervical pain (also related to a dropped head). Three subjects complained of itching eyes and one subject experienced vomiting after a session.

## Discussion: Feasibility of the Cursive Eye-Writing With EOL

This study showed that SPEM resulting in recognizable cursive eye-writing was feasible in a small group of ALS patients, with a level of performance comparable to control subjects. As observed in healthy subjects, and despite adaptive training, the learning rates and the capability to eye-write widely differed across ALS patients and across control subjects, without being able, at this stage, to determine whether such variability relates to idiosyncratic differences in perceiving the motion illusion, in cognitive or psychological traits, or in differences in motor control. From this and other eye data sets, we could not see any trend for an effect of age or education. Understanding the origins of the observed inter-individual differences is in itself a scientific issue for which separate studies are needed. What the present study points to, is the observation that ALS and control subjects do not differ much in their ability to master SPEM with EOL, with different, albeit consistent, profiles. In particular, those participants able to initiate and maintain SPEM for long durations, in early or late sessions, were also those able to master eye-writing so as to produce legible digits (Figure 6), and to write letters or small words at will.

In ALS participants, asthenia, motivation or anxiety are unlikely to account for inter-session variability in SPEM, as these remained stable across sessions. The limited sample size and missing data related to the ALS disability evaluation restricted the use of neuropsychological data to clinically assess underlying cognitive impairments. Nevertheless, we noticed that one ALS subject not improving its capability to generate SPEM was significantly cognitively impaired. Further, four of the five participants (A, B, J, L) with limited SPEM production at initial visits were diagnosed as apathetic versus none of the subjects immediately considered as good performers. Thus, the hypothesis of a frontal dysfunction as a limiting factor of EOL use can be raised, but should be confirmed on a larger population using more appropriate cognitive screening tools for patients with motor impairments, such as the Edinburgh Cognitive and Behavioral ALS screen (ECAS, Niven et al., 2015).

The observation of similar inter-individual differences in ALS and control participants suggests they cannot be exclusively related to the disease and its consequences, and are a general feature. That ALS and control participants exhibited similar performance in the digit recognition experiment indicates that eye-movement control was not deeply affected in ALS subjects, despite them being significantly impaired in other motor modalities (see Table 1). This finding is in line with studies showing that oculomotor neurons are relatively spared in ALS, as compared to other motor neurons (Brockington et al., 2013; Nijssen et al., 2017), which was the rationale for using eye-controlled communication devices with ALS participants. Nevertheless, there is evidence that this likely is a resistance rather than a complete sparing of oculomotor functions, as a range of eye-movements disorders occur at late stages (Donaghy et al., 2011; Moss et al., 2012; Gorges et al., 2015).

As the ALS participants had to be able to come on-site to follow the protocol on a short time period, they necessarily had moderate levels of disability, as stated by the mean ALSFRS score. This population was thus not representative of ALS patients in a compelling need for an ACD. However, a recent study (Londral et al., 2015) demonstrated the positive impact of ACD on the quality of life of ALS patients and caregivers at an early stage, giving patients the opportunity to improve their communication skills as the disease developed. Unfortunately, it was not possible to follow-up the ALS subjects to evaluate whether participating to this study had an impact on their quality of life at later stages. Although using EOL probably depends on the ALS progression and the level of disability, the effects on an initial training at later stages remain to be established. In this regard, we note that a recent longitudinal study involving oculomotor tasks (Proudfoot et al., 2016) suggests that higher-level oculomotor functions remain relatively spared throughout the course of the disease, despite progressive increasing disabilities. However, this later study involved making saccades toward visual targets; SPEM or the voluntary generation of SPEM was not evaluated. Whether training the oculomotor system of ALS has positive effects at an early stage (neuroprotection, improved motor control allowing the use of eye-based ACD at a later stage) remains an open issue.

As all video-based eye-trackers, EOL is safe and did not induce serious adverse events. As with eye-based ACDs, EOL requires efforts to master and control eye-movements in an unusual way, often inducing fatigue during the training phase. The use of a flickering background as a substrate on which SPEM can be generated did not specifically elicit complaints, since the contrast of the display was lowered once the motion illusion was well identified, and because the flickering frequency remained relatively low. A limitation worth mentioning is that the head-mounted eye-tracker used here had a sampling rate limited to 60 Hz, which appeared too low to provide good renderings of SPEM.

There are limitations to the use of EOL that must be addressed. First, the criteria used to include ALS participants in this study (see Study Design) already indicate that not all ALS subjects can use EOL, and that using EOL may become difficult or impossible as the disease evolves. In addition, this study only evaluated the production of digits, not the production of words or of on-line communication for significant durations, or in everyday life. The need for training, and the risk that training does not necessarily lead to mastering eye-writing, limits the possibility of routinely proposing EOL as a communication tool. In this regards, the lack of understanding of the origins of the observed inter-individual differences prevents predicting who could benefit from EOL.

To date, EOL should probably not be considered an alternative to existing ACDs, but rather as an adjunctive tool providing a new creative space. It could thus be added to classical ACDs using fixed items that subjects fixate and select in succession, as is the case when writing by selecting a letter from the alphabet. Nevertheless, in contrast with other systems, EOL brings both creativity and personal expression, allowing individuals to initiate eye-movements that reflect their own actions and states, which may ease communication with beneficial psychological consequences, both for ALS patients and caregivers.

In this study, no attempt was made to use sophisticated algorithms to recognize eye-written characters, although smoothing allowed improving the rendering of eye-written digits. We did develop algorithms that can reliably identify eye-written characters from trained healthy subjects, as well as the identity of the writers (Diard et al., 2013; Chanceaux et al., 2014) which could ease the use of EOL in the future.

## Ethics Statement

The study was carried out in accordance with the Declaration of Helsinki and was approved by all relevant ethics committees and national regulatory authorities (CPP n°14942 IDF V; ANSM2014-A00392-45). All participants gave informed consent (written consent whenever it was possible).

## Author Contributions

TL, JM, LL, YV, and JL designed and wrote the protocol. JM, JL, and MV-M ran the experiments. JM and JL analyzed the behavioral results. AF and LL ran and analyzed the neuropsychological tests. TL, GB, NLF, MdMA, PFP, and FS recruited and examined the patients. TL, JM, and JL wrote the manuscript. LL hosted the study and facilitated the recruitment of the patients.

## Conflict of Interest Statement

The authors declare that the research was conducted in the absence of any commercial or financial relationships that could be construed as a potential conflict of interest.

## Footnotes

1 http://eol.scicog.fr/accueil/accueil_ang.html

2 https://clinicaltrials.gov/ct2/show/NCT02313402
