# Supplementary material for: Cursive Eye-Writing With Smooth-Pursuit Eye-Movement Is Possible in Subjects With Amyotrophic Lateral Sclerosis
Source: Front Neurosci. 2019 May 29;13:538. doi: 10.3389/fnins.2019.00538 (PMC6548885; doi:10.3389/fnins.2019.00538)
Supplement: Supplementary file 1 [file Table_1.docx]

**Supplementary table 1.** Mean (and SD) values for the quality of fixation and pursuit.

|  | Quality of Fixation | Quality of Pursuit |
| --- | --- | --- |
| First-half (FH) | 14.44 (17.44) | 1.13 (0.018) |
| Second-half (SH) | 13.34 (10.17) | 1.12 (0.014) |

The quality of fixation and of tracking a visible target were quantified as described in section 2.6.1.

**Supplementary table 2.** Mean (and SD) values for the time spent (%) generating SPEM with EOL for the first (2-3: FH) and last (5-6: SH) sessions.

|  | EOL (average) | EOL (best record) |
| --- | --- | --- |
| First-half (FH) | 63.99 (17.22) | 79.04 (17.52) |
| Second-half (SH) | 64.01 (14.46) | 81.61 (12.66) |
|  |  |  |

The duration and proportion of SPEM, relative to fixation, saccades and blinks generated with EOL during a run (30 to 60 seconds) were computed using the criteria defined in section 2.6.1: Smooth pursuit is any eye-trace comprising at least a cluster of 30 successive samples (500 ms of recording) spaced by less than 50 pixels to avoid including saccadic eye-movements, whose standard deviation was larger than 10, to avoid including fixational eye-movements. The cumulated SPEM duration is expressed in % of the run duration.

**Supplementary table 3.** Mean (and SD) values (%) for Motivation and Tiredness.

|  | Tiredness | Motivation |
| --- | --- | --- |
| First-half (FH)  Session 1-3 | 25.13 (26.68) | 86.05 (18.85) |
| Second-half (SH)  Sessions 4-6 | 27.26 (24.81) | 88.73 (12.61) |

**Supplementary table 4.** Neuropsychological evaluations

| Subject | age | Sex | Education (years) | | Anxiety (HAD) | | Depression (HAD) | | Behavior  FBI | | | Apathy  (Starkstein scale) | | Global cognitive efficiency  (MDRS) | | | | Identi  -fication of emotion (from SEA) | | | Theory of Mind  (from SEA) | | |  |
| --- | --- | --- | --- | --- | --- | --- | --- | --- | --- | --- | --- | --- | --- | --- | --- | --- | --- | --- | --- | --- | --- | --- | --- | --- |
| A | **62** | M | 15 | | n | | **P** | | | *N.A.* | | | **P** | | **P** | | | | **P** | | | **P** | | |
| B | **66** | M | 12 | | n | | n | | | **P** | | | **P** | | n | | | | n | | | n | | |
|  |  |  |  | |  | |  | | |  | | |  | |  | | | |  | | |  | | |
| C | **51** | F | 9 | | n | | n | | | *N.A.* | | | n | | n | | | | n | | | n | | |
| J | **20** | M | 14 | | n | | n | | | n | | | **P** | | n | | | | n | | | n | | |
| L | **53** | F | 12 | | n | | n | | | n | | | **P** | | n | | | | n | | | n | | |
|  |  |  |  | |  | |  | | |  | | |  | |  | | | |  | | |  | | |
| D | **61** | F | 12 | | n | | n | | | n | | | n | | n | | | | n | | | n | | |
| E | **49** | M | 17 | | n | | n | | | n | | | n | | n | | | | n | | | n | | |
| F | **50** | M | 17 | | n | | n | | | *N.A.* | | | n | | n | | | | n | | | **P** | | |
| G | **66** | M | 17 | | n | | n | | | n | | | n | | n | | | | n | | | **P** | | |
| H | **71** | M | 20 | | n | | n | | | n | | | n | | n | | | | n | | | n | | |
| I | **74** | M | 17 | | n | | n | | | n | | | n | | n | | | | n | | | n | | |
| K | **59** | M | 10 | | n | | n | | | n | | | n | | n | | | | n | | | n | | |
| **N.A. = not available ; n=normal score; P= pathological score** | | | |  | |  |  |  | | |  | | | | |  |  | | |  | | |  | |
